# A new biomechanical model of the mammal jaw based on load path analysis

**DOI:** 10.1242/jeb.247030

**Published:** 2024-09-30

**Authors:** Alec T. Wilken, Julia A. Schultz, Zhe-Xi Luo, Callum F. Ross

**Affiliations:** ^1^ The University of Chicago, Department of Organismal Biology and Anatomy, 1027 E 57th Street, Chicago, IL, 60637, USA; ^2^ Rheinische Friedrich-Wilhelms-Universität Bonn, Section Paleontology, Institute of Geosciences, 53115 Bonn, Germany

**Keywords:** *Didelphis*, Trabecular bone, Finite element analysis, Mandible

## Abstract

The primary function of the tetrapod jaw is to transmit jaw muscle forces to bite points. The routes of force transfer in the jaw have never been studied but can be quantified using load paths – the shortest, stiffest routes from regions of force application to support constraints. Here, we use load path analysis to map force transfer from muscle attachments to bite point and jaw joint, and to evaluate how different configurations of trabecular and cortical bone affect load paths. We created three models of the mandible of the Virginia opossum, *Didelphis virginiana*, each with a cortical bone shell, but with different material properties for the internal spaces: (1) a cortical-trabecular model, in which the interior space is modeled with bulk properties of trabecular bone; (2) a cortical-hollow model, in which trabeculae and mandibular canal are modeled as hollow; and (3) a solid-cortical model, in which the interior is modeled as cortical bone. The models were compared with published *in vivo* bite force and bone strain data, and the load paths calculated for each model. The trabecular model, which is preferred because it most closely approximates the actual morphology, was best validated by *in vivo* data. In all three models, the load path was confined to cortical bone, although its route within the cortex varied depending on the material properties of the inner model. Our analysis shows that most of the force is transferred through the cortical, rather than trabecular bone, and highlights the potential of load path analysis for understanding form–function relationships in the skeleton.

## Teaser

**Summary:** Load path analysis of a mammalian mandible allows precise mapping of force transfer routes through the cortical bone. This novel application of an engineering method offers biomechanical insight into skeletal form–function relationships.

## INTRODUCTION

Tetrapod jaws exhibit a wide range of variation in morphology in response to diverse functional demands, as well as developmental and evolutionary constraints. Functional explanations of this morphological diversity have focused on how mandibles amplify force, through metrics such as mechanical advantage, measured using lever mechanics (e.g. Westneat, 2003; Morales-Garcia et al., 2021; Tseng et al., 2023) or how mandibles resist *in vivo* forces during feeding, measured by finite element modeling (FEM) (e.g. Porro et al., 2011; Tseng et al., 2011; Panagiotopoulou et al., 2020; Smith et al., 2021). Lever mechanics and three-dimensional finite element analysis (FEA) have improved our understanding of how mechanical demands have shaped the evolution of mammalian mandibles (Crompton, 1963; Crompton and Hylander, 1986; Gill et al., 2014; Reed et al., 2016; Grossnickle, 2017; Smith et al., 2021, 2023). However, much less effort has been devoted to understanding what we argue may be the primary function of mandibles: force transfer from muscle attachment areas to bite points and jaw joints (Ross and Iriarte-Diaz, 2019).

Some of the earliest work on form–function relationships in the skeleton focused on force transfer through bones. Indeed, the archetypal mechanical explanation for the trajectorial arrangement of trabeculae in bones is that cancellous bone morphology is constructed so as to resist the stresses to which it was subjected with the minimum amount of material (Cullman, 1866; von Meyer, 1867; Wolff, 1869; Skedros and Baucom, 2007; Sinclair et al., 2013; but see Bertram and Swartz, 1991; Cowin, 2001). In contrast, Wilhelm Roux, in his theory of *Entwicklungsmechanik* (Roux, 1881, 1885, 1895), postulated that tubular bones form when the more compact external cortical bone hypertrophies in response to loads, the cortical bone can unload the inner trabecular bone as a trade-off, leading to atrophy of trabeculae. According to Roux, cancellous bone spreads resistance to loads across a larger area than would be necessary for a simple piece of compact bone under pure compression or tension, allowing the weight of the bone to be reduced. Under Roux's model, the cortical bone is the primary load path for transfer of force whereas trabecular bone is a less important trajectory (see the review of Roux's work by Walkhoff, 1902).

Shortly after the initial application of the trajectorial theory to trabecular bone in the femur (Cullman, 1866; von Meyer, 1867; Wolff, 1869; Bertram and Swartz, 1991), Walkhoff applied it to the trabecular bone structure of primate mandibles, seeking ‘to prove that Roux's observations are in fact the foundation for the evaluation of the variable jaw structure and jaw form of primates’ (Walkhoff, 1902: p. 210). Like Roux, Walkhoff hypothesized that, when forces and stresses are constant, the trabeculae that are highly stressed will be retained, producing trajectories: continuous trains of trabeculae that develop where there is complete constancy of ‘pressure direction’ (literally, *Druckrichtung*) (Walkhoff, 1902: pp. 213–214). Walkhoff used the then new X-ray radiography to identify trajectories in the mandibles of great apes, and he further hypothesized these trajectories would function to transfer force between muscle attachment areas, bite points, and jaw joints. Walkhoff's image of the trajectories in the ramus of adult *Pongo* became an iconic graphic representation of this trajectorial theory, for mandibles.

**Fig. 1.**
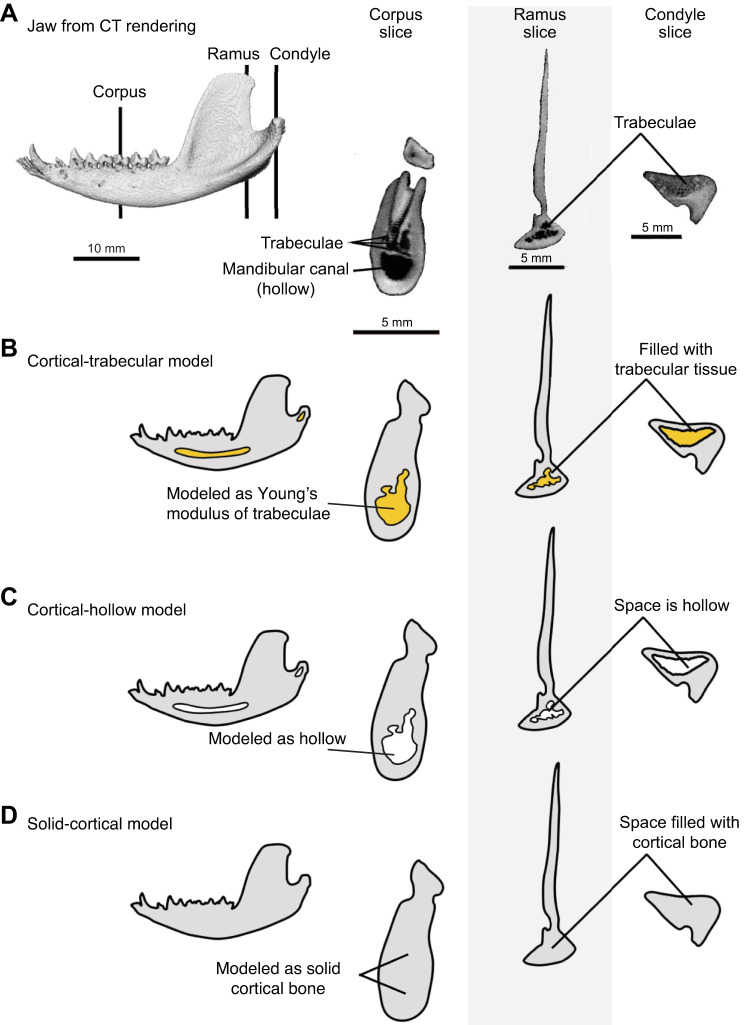
**Alternative modeling approaches for the interior space inside the cortical bone shell of the mandible of *Didelphis virginiana*.** This interior model includes the mandibular canal and the trabecular bone of the alveolar process, angular shelf and condylar process. Three different models are used to test hypotheses about the role of trabecular bone in the load path of the mandible. (A) CT rendering of a mandible of *Didelphis virginiana*, with coronal slices from the mandibular corpus (at m2), ramus (tip of coronoid process) and condyle (articular process), showing the internal geometry of the mandibular bone as revealed by µCT. (B) Cortical-trabecular model: the interior trabecular bone and anatomical space of mandibular canal are treated together as trabecular bone in a modular component assigned with the Young's modulus of bulk trabecular bone. Schematic jaw model with trabecular and anatomical spaces (left), corpus slice (middle), ramus slice (right) and condyle slice (far right). Intertrabecular spaces and anatomical spaces in yellow. (C) Cortical-hollow model: the trabecular bone (with its intratrabecular space), and mandibular canal are simplified and both treated as hollow spaces, within the cortical bone shell that is modeled with Young's modulus of cortical bone. (D) Solid-cortical model: all internal structures (including intertrabecular space and mandibular canal space) are treated as solid cortical bone and modeled with the Young's modulus of cortical bone.

Walkhoff distinguished between external ridges of cortical bone on the mandible – for example, external and internal oblique lines, endocondylar and endocoronoid ridges – from internal trajectories composed of trabecular bone. But limited by planar X-ray available during his time, he was not able to discriminate between the trabecular trajectories from the cortical bone using 2D radiographs. The exact morphological identities of these trajectories, as interpreted from his 2D radiographs, were questioned by others very early on (Görke, 1904; Todlt, 1904). Recent advances in CT scan imaging have now made it possible to identify the morphology of these putative trajectories, more precisely (Fig. 1).

Moreover, complementary advances in finite element modeling (FEM) have allowed hypotheses about the transfer of force through biological structures to be tested. For example, on the basis of qualitative correspondence between surface features and areas of high strain magnitude, Panagiotopoulou et al. (2020) hypothesized that robust external features of the mandible – the medial prominence, endocondylar ridge and torus triangularis – might constitute the primary load path, which is the shortest, stiffest route of force transfer, between bite points, muscle attachment areas and jaw joints. A corollary of this is that the trabecular bone structures of the mandible, historically assumed to represent the trajectories of load, are less important in force transfer than cortical bone; a hypothesis supported by the fact that trabecular bone is roughly 20–30% less stiff (and thus less efficient in force transfer) than the surrounding cortical bone (Beaupré et al., 2000). Clearly, we need quantitative estimates to objectively determine whether load paths map to specific morphological components of different material properties of the mandible, and to locate the load path within the mandible.

Here, we present a quantitative framework for mapping load paths and quantitatively assessing their relationships to both the surface features and the interior cortical-trabecular geometries of the mandible. We demonstrate this method by quantifying the location of the primary load paths through the mandible of the Virginia opossum, and asking whether these load paths pass through cortical or trabecular bone, and whether their location is related to external ridges on the mandible.

Most parts of a continuous structure play a role in force transfer; however, some areas carry more of the load than others (Wang et al., 2017). Load paths are the shortest, stiffest routes from the loading points (e.g. muscle attachments) to the support constraints (e.g. temporomandibular joints) and bite point. And they are the primary paths of force transfer through a structure (Kelly et al., 2011; Wang et al., 2017; Zhao et al., 2021). The load paths and load bearing elements in a structure may be identified and optimized through load transfer analysis (Zhao et al., 2021). Load paths are defined by five rules (Singh, 1996): (1) load paths begin on the point of load application and end on load reaction boundaries (in the mandible, the jaw elevator muscle attachment sites, the bite points and the jaw joints); (2) under uniform loading, load paths spread as evenly as possible and cover the whole structure; (3) load paths never cross each other; (4) load paths may diverge no more than 35 deg around a geometric discontinuity (e.g. a hole) because this would create stress concentrations that could weaken or break the structure; and (5) curvature in a load path is associated with creation of shear and bending moments. Taken together, these principles constitute a coherent approach to load path analysis.

**Fig. 2.**
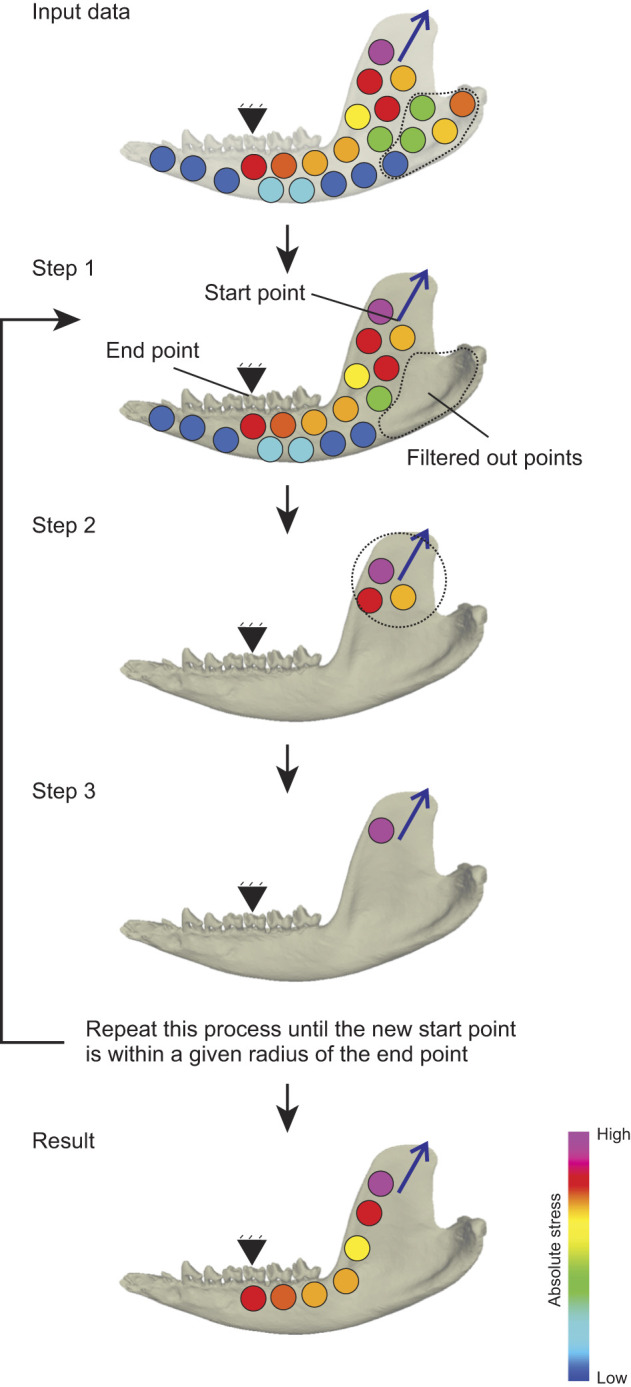
**Graphical representation of load path modeling of the mandible of *Didelphis virginiana*.** Load paths are calculated using an iterative process that seeks out the continuous series of highest loaded elements from the starting point of mandibular adductor muscle attachment (blue arrow on coronoid process) to the boundary constraint (in this case, the bite point). Input data: elements (colored circles) with associated coordinates and load values. Step 1: filter out points further away from the end point than starting point is. Step 2: filter out points outside a given radius of the start point (dotted circle). Step 3: select the remaining point with the highest absolute stress. Redefine the start point. Then repeat this process until the new start point is within a given radius of the end point. The result is a string of elements that comprise the primary load path. The iterative process is repeated until the calculated load path reaches the bite point (end point). Separate load path calculations are performed for the path from each muscle attachment area, the bite point and bilateral jaw joints. Schematic symbols: triangle, the bite point; blue arrow, the vector of jaw adductor muscle.

There are several methods to calculate a load path (Shinobu et al., 1995; McConnel and Brown, 2011; Kelly et al., 2011; Wang et al., 2017; Zhao et al., 2021). Each load path metric has its own strengths and weaknesses (Zhao et al., 2021), which must be considered before applying them to mandible biomechanics. The most commonly employed methods include plotting the direction of the first principal stress, transferred force, the U* index and absolute stress. The direction of the first principal stress is useful in load path analysis because the first principal stress vectors are parallel to the load path (Bassandeh, 2012). Plotting the first principal stress vectors to illustrate the load path is a common practice in engineering and is computationally most feasible; however, this method works best under unidirectional loading and as such it is of limited value in the more complex loading environments commonly found in biological situations (Zhao et al., 2021). The transferred force approach to load path analysis is accomplished by deleting an element from the structure and evaluating the deletion's impact on the reaction force at boundary constraints. This is an intuitive but time consuming method (Zhao et al., 2021). Another key metric of load path analysis is the U* index, which is defined as a dimensionless measurement of load transferability introduced by Shinobu et al. (1995). The U* index analyzes load transfer via strain energy differentials to calculate the internal stiffness of each point (Shinobu et al., 1995). Under U* theory the load path is then the negative contour of U* values. The U* index is computationally intensive but is not prone to artifacts of complex geometries or loading conditions (Shinobu et al., 1995; Zhao et al., 2021). Here, we calculate and visualize the load path using the absolute stress method (Fig. 2), which uses the principal stress with the highest magnitude at each element to identify the load path. This method is effective in showing load paths in complex loading environments, such as those of mandibles and is also computationally efficient (Zhao et al., 2021). Load paths have been used to study and optimize automobile design for collisions, determine optimal trajectories of fibers in sails, and map the path of stress in the frame of helicopters (Sakurai et al., 2003; Bassandeh, 2012; Kelly et al., 2019), among other engineering structures. These analyses identify the most load bearing parts of a structure and allow for optimization of materials.

Understanding load paths in mandibles is crucial because force transfer is an important function of the jaw. Load path analysis is a promising bridge between understanding patterns of loading and internal and external bone morphology, bringing trajectory theory into the 21st century. Synthesizing load path analysis and the trajectory theory refines the long-standing question of the morphological identity of trajectories of the interior cortical-trabecular structures into a simple trade-off: will a load path favor a route through trabecular bone which is less stiff, but could be a potentially shorter route, or a route through cortical bone which is stiffer but can be potentially longer?

Here, we apply load path theory to investigate the roles of trabecular and cortical bone in force transfer through the mandible of the Virginia opossum *Didelphis virginiana*. We selected the opossum as a model organism to study load paths because *Didelphis* is an omnivore, its mandible consistently falls in the center of mammal jaw morphospace (Bhullar et al., 2019; Morales-Garcia et al., 2021; Tseng et al., 2023) and its mandibular morphology is generalized, so is often used in comparative studies of early mammal evolution (Crompton, 1995; Luo, 2007). For these reasons, *Didelphis* has long been a model species in feeding biomechanics of mammals (Crompton and Hiiemae, 1970), and the motor function of *Didelphis* and related *Monodelphis* is relatively well studied (Thomason et al., 1990; Crompton, 1995; Lieberman and Crompton, 2000; Bhullar et al., 2019; Stilson et al., 2023). Hence, the mandible of *Didelphis* can be modeled with reasonable accuracy.

We designed models to answer a simple question: do the load paths of the mandible run through trabecular bone, cortical bone, or both? To perform this experiment, we created three finite element models (Fig. 1). (1) The cortical-trabecular model includes a cortical bone shell but the interior of mandible (including the intratrabecular space and anatomical space of the mandibular canal) is filled by material with properties of trabecular bone (Fig. 1B). (2) The cortical-hollow model consists of a cortical bone surrounding an empty interior space that represent both the mandibular canal plus the space for trabecular bone (Fig. 1C) and this model simplifies by removing the trabeculae. (3) The solid-cortical bone model consists of a completely solid cross-section made of cortical bone; a model made by filling the inner ‘space’ with material with the material properties of cortical bone (Fig. 1D). The latter models have been used by several recent studies (Gill et al., 2014; Lautenschlager et al., 2023).

In mammalian mandibles, muscle forces must be transmitted from the jawbone through the periodontal ligament around the tooth roots, to reach the bite point. The effects of periodontal ligament in the alveolar sockets on mandible loading have long been of research interest. Early descriptions of mandibular trajectories included a ‘trajectorium dentale’, which travels through the alveolar process (Walkhoff, 1902). The possible presence of the trajectorium dentale implies that periodontal ligament would influence the stiffness of the alveolar process and could be relevant to the accurate mapping of load paths in the mandible. Daegling et al. (1992) demonstrated that periodontal ligament affects the transmission of stresses between alveolar sockets and the tooth roots. Some studies have shown that omitting periodontal ligament from FEA models can produce significant errors in strain values in the mandible (Gröning et al., 2011, 2012; Gröning and Fagan, 2012). Other studies found that including the periodontal ligament in a finite element model (FEM) results in an increase in strain in the alveolar process of the mandible while the rest of the mandible is largely unaffected (Wood et al., 2011; Abraha et al., 2019), consistent with the postulation that the tooth socket raises stress only locally (Daegling et al., 1992; McGraw and Daegling, 2020; Chen and Chen, 1998). From these considerations, it is important to assess the impact of the periodontal ligament on load path calculations of mammal mandibles. Therefore, inclusion or exclusion of periodontal ligament should be an important part of sensitivity analyses of mandible load paths.

## MATERIALS AND METHODS

### Materials

A dry skull of an adult *Didelphis virginiana* [RV670, University of Chicago Research Collections (UCRC), housed in Department of Organismal Biology and Anatomy] was µCT scanned (Wilken et al., 2024). The specimen was scanned at a voltage of 200 kV, with a voxel size of 85.997 µm, on GE Phoenix v|tome|x dual tubes 180kv/240kv scanner in the University of Chicago (https://luolab.uchicago.edu/paleoct/).

### Methods

#### Model creation

All parts of the the mandible (outer shell, internal spaces, trabecular bone) were 3D-modelled by manually segmenting slices of the CT scanned skull using the software package Avizo v.9 (Thermo Fisher Scientific, Waltham, MA, USA). Trabecular bone was distinguished from cortical bone based on structure and gray scale value. Trabecular bone shows a sponge-like structure in CT scans (unsorted pattern, medium to light gray values with dark hollow spaces) that distinguishes from solid cortical bone (homogeneous pattern sometimes layered, universal gray values appearing as medium or light gray; see Fig. 1A). These models were then imported into Geomagic v.17 (3D Systems, Rock Hill, SC, USA) where problematic elements, such as intersecting triangles, small spikes and holes and floating triangles, were removed, and the models were aligned to the anatomical coordinate system used in other studies (e.g. Panagiotopoulou et al., 2017, 2020; Smith et al., 2021, 2023). The models were then imported into Strand7 (Beaufort Analysis, Sydney, NSW, Australia) where they were solid meshed for finite element modeling using four-noded tetrahedra.

For modeling the interior material of the mandible, the trabecular space and mandibular canal were treated together as an internal modular component (by virtually wrapping the 3D models of both trabecular and hollow spaces using the wrapping tool of 3-Matic (Materialise, Leuven, Belgium), which then was inserted into the mandible (outer shell), for the whole mandible model to be solid meshed in Strand7. This modular component was either modeled as a space filled with trabeculae (Fig. 1B), or as a hollow cavity (Fig. 1C) or as solid bone (Fig. 1D). Three alternative mandible models were constructed: a cortical-trabecular model (534,159 bricks), in which the outer shell was assigned the material properties of cortical bone and the internal modular component was assigned the material properties of trabecular bone; a cortical-hollow model (633,419 bricks), in which the modular component was treated as an empty cavity inside the shell with material properties of cortical bone; and the solid-cortical bone model (310,715 bricks), in which the modular component was treated as solid cortical bone like the outer shell.

The soft tissues of the unfused mandibular symphyses, including an anterior fibrocartilaginous pad and posterior cruciate ligaments (Scapino, 1981; Lieberman and Crompton, 2000), were modeled using beam elements. Material properties (Dataset 1) of these beam elements were derived from Beaupré et al. (2000) for cartilage and Kupczik et al. (2007) for sutural ligaments. Sutural ligaments were modeled as tension-only cutoff bars.

Periodontal ligament was not included in these three mandible models, but we performed a separate sensitivity analysis to compare a model lacking a periodontal ligament, namely the tooth roots are treated as if fused with the root socket, with a model that includes not only a periodontal ligament, but also varying material properties for tooth roots and the alveolar socket wall. The material properties for teeth (including roots) and periodontal ligament follow Abraha et al. (2019) (Dataset 1). Cortical bone material properties were derived from Dechow et al. (2017) and trabecular bone properties from Panagiotopoulou et al. (2017) (Dataset 1).

#### Muscle modeling

Muscle forces were calculated from review of the literature (Turnbull, 1970; Hiiemae and Jenkins, 1969; Lautenschlager et al., 2017), DiceCT and dissection. Muscle origin and insertion sites were used to calculate muscle volume using a frustum *sensu* Sellers et al. (2017) with Eqn. 1:
(1)

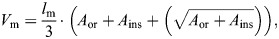
where *V*_m_ is muscle volume, *l*_m_ is the muscle length, *A*_or_ is the muscle origin site area and *A*_ins_ is the muscle insertion area (Sellers et al., 2017). This volume was then used to calculate PCSA (physiological cross-sectional area) according to Eqn 2 (Sacks and Roy, 1982):
(2)

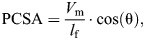
where *l*_f_ is muscle fiber length and θ is muscle fiber pennation angle (Sacks and Roy, 1982). Muscle force was then calculated from PCSA using Eqn 3 (Gans, 1982):
(3)

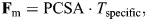
where **F**_m_ is muscle force and *T*_specific_ is specific tension of the muscle. Muscle fiber length was assumed to be 2/3 of muscle length and pennation angle was assumed to be 0 deg *sensu* Cost et al. (2020). The value for specific tension was chosen to be 0.3 N mm^–2^ (Hieronymus, 2006). Finally, muscle forces were scaled using *in vivo* EMG data reported by Lieberman and Crompton (2000). We modeled jaw muscle activity during the slow close phase of the power stroke of an opossum chewing on beef. BoneLoad was used to apply muscle forces over the areas of attachment on cranium and mandible (Grosse et al., 2007; Davis et al., 2010). We calculated muscle forces for the anterior temporalis, posterior temporalis, deep masseter, superficial masseter, medial pterygoid and lateral pterygoid. To demonstrate the sensitivity of load paths to modeling different external loading regimes, we also applied loading regimes of the power stroke of an opossum chewing on bone, and chewing on beef, a triplet I (wherein the working side posterior temporalis, the balancing side medial pterygoid and the balancing side superficial masseter are active; Weijs, 1994) and full muscle activation. Muscle modeling parameters are given in Dataset 1.

#### Finite element analysis

The constraints were based on *in vivo* kinematics (Stilson et al., 2023). One node was constrained on each jaw joint and on the bite point (left m2). The working side jaw joint and the bite point were constrained against superior–inferior translations, anterior–posterior translations and lateral (but not medial) translations. To model the sensitivity of load paths to differing bite points we also ran models where p2 and the last lower tooth, m4, were constrained in the same manner. The balancing side jaw joint was constrained against superior–inferior translations and anterior–posterior translations.

#### Load path analysis

The load path is the stiffest, shortest route from a point of force application (e.g. a muscle attachment) to a boundary constraint such as the bite point or a jaw joint (Fig. 1) (Kelly et al., 2011; Wang et al., 2017; Zhao et al., 2021). Load paths were calculated using the absolute stress load path method (e.g. Zhao et al., 2021). Absolute stress is defined by Eqn 4 (Zhao et al., 2021):
(4)

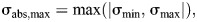
where σ_abs,max_ is absolute stress, σ_min_ is the second principal (minimum compressive) stress and σ_max_ is the first principal (maximum tensile) stress (Zhao et al., 2021). Under the absolute stress load path theory, the load path is defined as the continuation of maximum absolute stress values between the point of force application and the boundary constraint. To calculate the load path, we developed an algorithm in R (Fig. 2). The algorithm acts on a data frame containing the *x*, *y*, *z* coordinates and absolute stress value for every brick element in the FEM. Coordinates are then given for the starting point of the load path. BoneLoad applies forces across the entire muscle attachment site (Grosse et al., 2007; Davis et al., 2010), but we used the centroid coordinates of the muscle attachment as the start point for the load path calculation. We performed a sensitivity analysis on how changing the start point at different borders of the muscle attachment site and found that load path results are relatively robust to different start points (Fig. S1), making the centroid coordinates a pragmatic representation of the muscle attachment area. The algorithm then filters out any brick element that is not closer to the end point than the starting point, ensuring that the calculations estimate the load path to the end point – the specific boundary constraint being analyzed (Fig. 2: step 1). Next, points outside a radius of 1 mm from the start point are filtered out to find the bricks closest to the start point. Finally, the algorithm selects the brick with the maximum absolute stress out of the remaining bricks (Fig. 2: step 2). The process is then iteratively repeated until a brick is chosen that is within 1 mm of the end point (Fig. 2: step 4). Load paths were then plotted and visualized on mandible models (Fig. 2: results) using Maya 2020 (Autodesk, Inc., San Rafael, CA, USA). Because this load path methodology is stress based, models with large amounts of excessively distorted elements could produce artifacts in load path geometry. We examined the distribution of brick aspect ratios to ensure the models in this study would not be susceptible to such errors (Fig. S2). For ease of interpretation, load paths were only calculated for the temporalis and masseter muscles, although models were loaded with forces of more muscles depending on the load case. We believe that, given that the temporalis and masseter muscles attach on the superior and inferior extremes of the ramus, this simplification still captures the relevant variation in load path geometry.

Load paths can be compared using three criteria: uniformity, continuity and consistency (Zhao et al., 2021), of which the consistency criterion is more relevant for this study and most effective. Uniformity describes force transfer along the length of the load path and how constant that force transfer is (Zhao et al., 2021; e.g. Fig. S3). Continuity describes the curvature of the load path compared against an idealized load path (Zhao et al., 2021). Consistency describes the convergence of multiple load paths (Zhao et al., 2021). Deploying these criteria in a biological context requires consideration of their biological relevance.

Continuity is the least plausible load path criterion to apply in a biological context. Continuity requires an *a priori* concept of an ideal load path. A difficulty with this criterion is that it depends on prior knowledge of the geometry of an ideal load path in a mandible before we can apply this criterion. Moreover, there could be many different adaptive peaks of load path morphology in any given skeletal element. It is beyond the scope of this paper to resolve this.

The uniformity criterion can also be difficult to apply to musculoskeletal systems. We presently cannot hypothesize a scenario wherein the magnitude of the internal forces being transmitted in a bone are optimized by natural selection. We can further demonstrate that, for our purposes to discriminate among alternative mandible models of interior structure, it is also uninformative (Fig. S3). To calculate load path uniformity for comparison, forces in each brick element along the load path were derived from element node forces in Strand7. Only the forces parallel to the load path are transmitted along the load path (Zhao et al., 2013, 2021; Fig. S4), with force vectors orthogonal to the load path generating shear and bending moments about the load path. The element node force vectors were projected on to the local load path vector (Fig. S4A), thereby resolving the node force vectors on the load path into those components along and perpendicular to the local load path. None of the three models of interior bone configuration is any better fit for the uniformity of force transfer. Given these epistemological considerations, we quantified uniformity (Fig. S3), but only report these results in Fig S3.

Consistency, however, ought to be related to morphology. For example, given two load paths, if the two load paths do not converge on the same morphological location (i.e. are less consistent) this will result in two primary load bearing areas in a skeletal structure, as opposed to a more consistent couple of load paths (which converge on the same morphological location) which will imply one primary load bearing area. Assuming a mechanostat model of osteogenesis (e.g. Ruff et al., 2006), these situations could result in bony reinforcement in different regions of the skeletal structure.

To compare load path consistency, the minimum distance between each point in the load path of the masseter and the temporalis to a given boundary constraint was calculated. Because we know that the load paths must begin at separate locations (the attachment centroids of each respective muscle) and must end at the same location (the coordinates of the boundary constraint), we compared the minimum distance between the two load paths against the distance from the boundary constraint. Furthermore, we propose a null hypothesis of load path consistency wherein we expect the load paths to be linear and to become more consistent as they approach the boundary constraint. Under this model, load paths of these muscles are more consistent if these load paths plot under the slope of the null hypothesis, and load paths are less consistent if they plot over the slope of the null hypothesis. This analysis allows us to compare load path consistency quantitatively.

## RESULTS

### Model validation

**Fig. 3.**
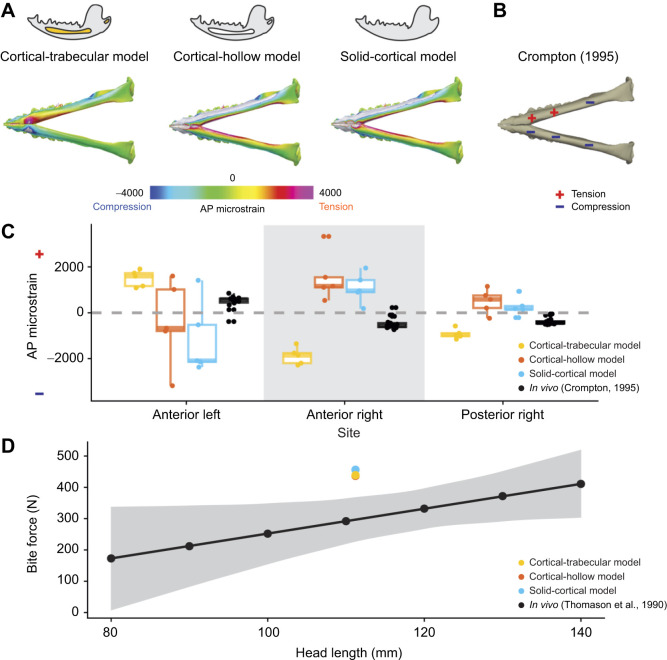
**Validation of alternative mandible models of different interior bone geometry.** Strains of three finite element models (FEMs) of mandible with different interior geometries are compared against *in vivo* strain and bite force values reported by previous studies. (A) FEMs of cortical-trabecular (left), cortical-hollow (middle) and solid-cortical (right) models and their strain regimes on ventral aspect of mandible (mandibles in ventral view). (B) *In vivo* strain of *Didelphis virginiana* reported by Crompton (1995) for comparison. Crompton recorded strain from three points on the bottom of each mandible using single element strain gauges aligned with the long axis of the base of the mandible. The color map bar shows the antero–posterior (AP) tension (axial tension along long axis) and compression (axial compression along the long axis). (C) AP strain data at three sites along the bottom of the mandible for the three FEMs of mandibular bone of different interior geometry; at all three sites, the cortical-trabecular FEM best matches the *in vivo* strain data from Crompton (1995). (D) *In vivo* measurement of bite force against head length in *D. virginiana* (Thomason et al., 1990, gray area outlines 95% confidence limit of biting force of the opossum). The cortical-trabecular FEM best matches the *in vivo* strain data, although it still overestimates strain magnitudes and bite force. The mandibular models with cortical-hollow or solid interiors are poorer fits to the available *in vivo* experimental data.

Finite element models (FEMs) of the three alternative models of interior geometries (cortical-trabecular, cortical-hollow and solid-cortical) of the mandible of *Didelphis* (Fig. 1) were compared against the *in vivo* bone strain magnitude data from *Didelphis* reported by Crompton (1995) and bite force magnitude data from *Didelphis* reported by Thomason et al. (1990). Crompton used uni-axial strain gauges to record strains from the inferior border of the mandible, so we compared anterior–posterior (AP) axial strain components (Fig. 3A) against the *in vivo* data reported by Crompton (1995) (Fig. 3B). The cortical-trabecular model best matches the *in vivo* AP strain regimes, showing compression throughout the ventral margin of the balancing side hemi-mandible and the posterior working side hemi-mandible, while showing tension anterior to the bite point (Fig. 3A,C). The cortical-hollow and solid-cortical models show relatively neutral loading along the ventral margin of both hemi-mandibles but show high compressive strains on the lateral surface of the working side hemi-mandible (Fig. 3A). Despite matching the overall strain regimes, the cortical-trabecular model overestimates strain magnitudes (Fig. 3C). Conversely, the cortical-hollow and solid-cortical models match the magnitudes of the *in vivo* strains better but are in opposite strain polarities. In other words, these two models are in completely different bending regimes than the bending regime reported from *in vivo* studies (Crompton, 1995). Comparison of FEM bite forces with published *in vivo* bite forces reveals that all models overestimate bite force by roughly 100 N, a 20.6% error (Fig. 3D). We believe, given the goals of this study, these are acceptable errors.

### Load path analysis

**Fig. 4.**
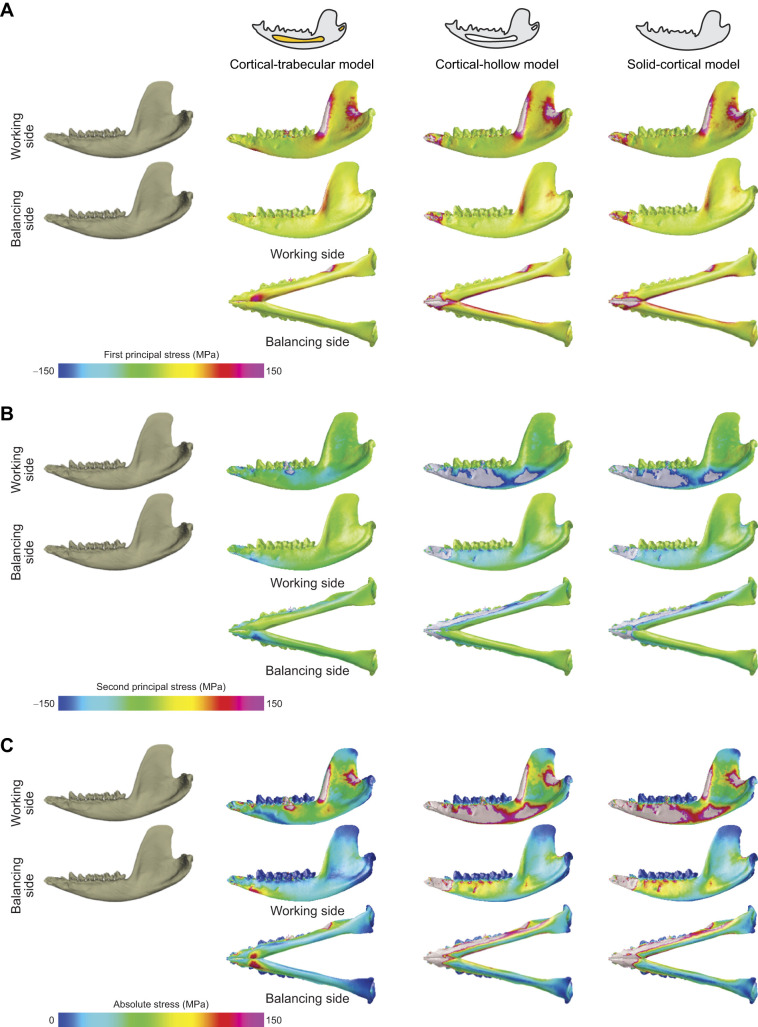
**Absolute stress magnitudes calculated for each model.** In A–C the upper row illustrates working mandible in left lateral view, the middle row is balancing mandible left–right flipped and mirrored as lateral view; lower row represents both working and balancing mandibles in ventral view. (A) Plots of first principal stress reveal regions with high tensile stress (redder colors). (B) Plots of second principal stress reveal regions with high compressive stress (dark blue). (C) Plots of absolute stress indicate highest absolute value of first or second principal stress. These absolute stress values are used to determine which regions are loaded to the highest magnitude and through which the most load is being transmitted through load path. Note high values of absolute stress in ridges on anterior and bottom borders of the ramus, and in a strip in the middle of the ramus above the peduncle of the condyle process.

Load paths were calculated using the absolute stress method (Fig. 4C). The cortical bone of the trabecular model experiences much lower stress magnitudes (both compressive and tensile) than the cortical-hollow and solid-cortical models (Fig. 4A,B). The masseter shelf is highly tensed and the angular process is compressed in all three models (Fig. 4; Fig. S4). The most anterior region of the mandibular symphysis is tensed (Fig. 4). Visual inspection of absolute stress distributions against load path geometry corroborates that the load path algorithm is outputting correct results.

**Fig. 5.**
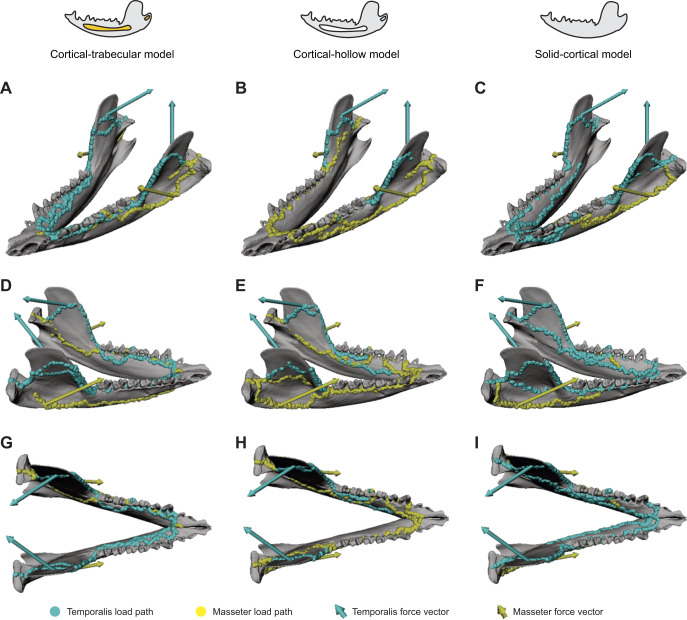
**Comparison of load path geometries among the three interior models and anatomical spaces inside the mandible.** (A–C) Left oblique view of load paths in the cortical-trabecular model (A), the cortical-hollow model (B) and the solid-cortical model (C). (D–F) Right oblique view of load paths in the cortical-trabecular model (D), the cortical-hollow model (E) and the solid-cortical mode (F). (G–I) Dorsal view of load paths in the cortical-trabecular model (G), the cortical-hollow model (H) and the solid-cortical model. In all three models, the load path is always on the periphery (cortical bone) of the bone.

In all three mandible models of interior structure, the ridge on the anterior edge of the ramus is the load path for temporalis muscle force into the mandibular corpus (Fig. 5). The masseter shelf is the load path for masseter muscle force into the corpus and to the condylar process (Fig. 5). The primary difference between the trabecular model and the other two models is on the balancing side hemimandible. In the trabecular model, the masseter load path travels on the ventral margin of the hemimandible until the mandibular symphysis (Fig. 5D). Moreover, in the trabecular model the load paths do not enter the trabecular bone, instead staying on the cortical bone on the periphery of the corpus (Fig. 5A,D,G).

**Fig. 6.**
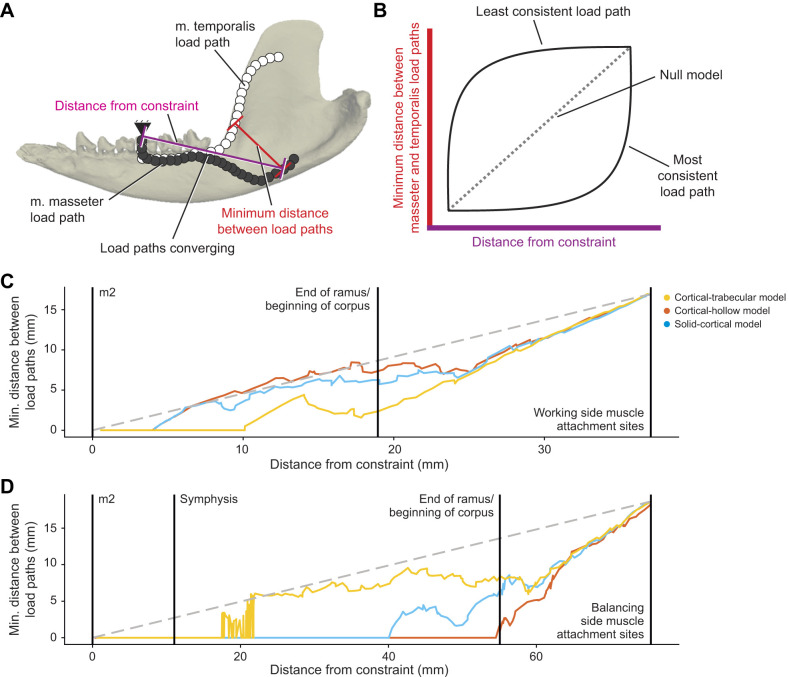
**Comparison of consistency of the three models of interior bone geometry treatment.** (A) Illustration of how distances are calculated for this analysis of load path consistency. Black arrowhead indicates the end point. (B) Conceptual schemes of three (least, null and most consistent) scenarios of load path consistency quantification, by minimal distance of temporalis and masseter load paths, mapped over load path distance from muscle attachment to bite points or jaw joints. (C) Consistency of load paths of working side from attachments of jaw muscles to the bite point (lower m2). (D) Consistency of load paths of the balancing side from attachment of jaw muscles to the bite point (m2). Gray dashed lines represent a null model of consistency. Space above the dashed line represents less consistent load paths, whereas space below the dashed line represents more consistent load paths than the null model. The cortical-trabecular model (yellow line) has the most consistent load paths from the working side jaw muscles to the bite point, but the least consistent load paths from the balancing side jaw muscles to the bite point.

Comparison of the three models of interior mandibular structure reveals differences in load path consistency (Fig. 6; Fig. S5). When comparing load path consistency to the bite point, all three models show greater consistency than expected from a null hypothesis (gray dashed line; Fig. 6C,D). The load paths from the working side jaw muscles on the cortical-trabecular model show the greatest consistency (Fig. 6C); however, the load paths from the balancing side jaw muscles in this model show the least consistency (Fig. 6D). These data illustrate the impacts of trabecular bone on load path optimization.

**Fig. 7.**
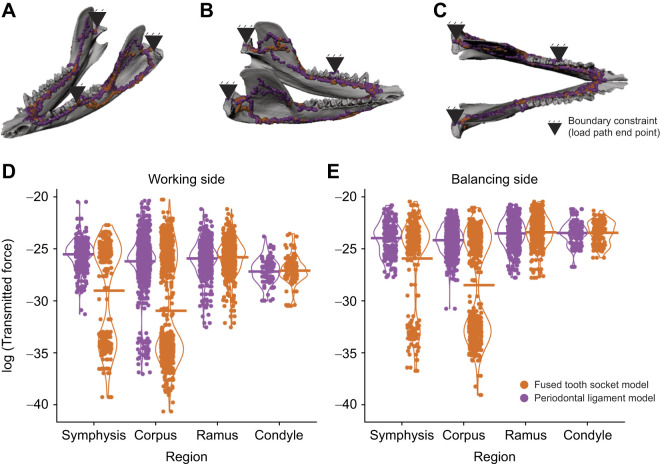
**The effects of modeling periodontal ligament on load path results.** (A–C) Load paths in the mandible in left lateral oblique view (A), right lateral oblique view (B) and dorsal view (C). (D) Comparison of forces transmitted along the load path in the fused tooth socket model (orange) and the periodontal ligament model (purple) on the working side hemi-mandible. (E) Comparison of forces transmitted along the load path in the fused tooth socket model (orange) and the periodontal ligament model (purple) on the balancing side hemi-mandible. The inclusion of periodontal ligament has negligible effects on the location/geometry of the load path but influences the forces transmitted along the load path.

Comparison of the model of fused tooth-roots and alveolar socket with the periodontal ligament model demonstrates that modeling periodontal ligament has limited effects on load path analysis of the mandible (Fig. 7). Including periodontal ligament has little effect on load path location and geometry, as the load paths in the fused tooth socket and periodontal ligament models rarely diverge from each other (Fig. 7A–C). Despite the similarities in load path geometry, including periodontal ligament in the mandible model increases uniformity of the load paths in the corpus and symphysis (Fig. 7D,E). In the ‘periodontal ligament’ model, transferred force is relatively high and constant in every region of the mandible (Fig. 7D,E). In contrast, the ‘fused tooth socket’ model shows highest transmitted force in the posterior regions of the mandible and every region has a bimodal distribution of high and low transmitted force along the load path in the corpus and symphysis (Fig. 7D,E). These data illustrate how force transfer along the load path changes in different regions of the mandible.

**Fig. 8.**
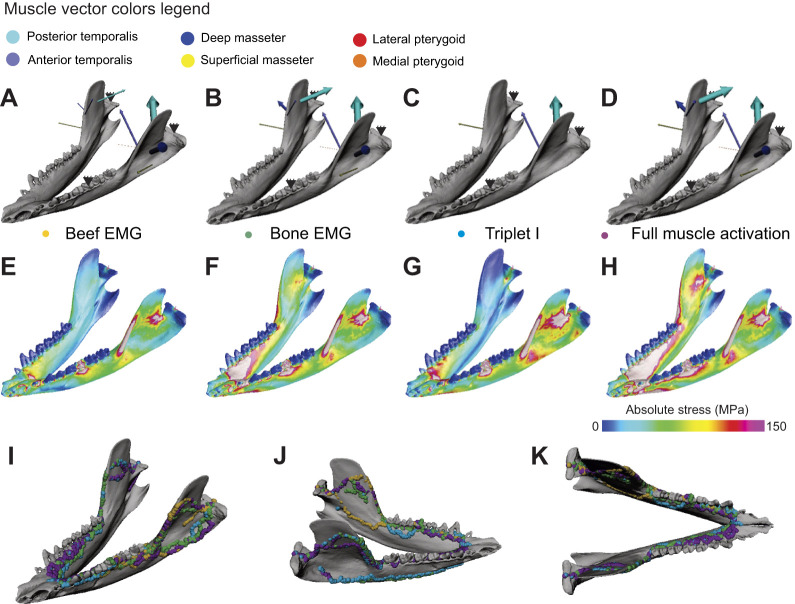
**Sensitivity analysis of the effect of loading regime on load path geometry on the cortical-trabecular model.** (A–D) Left oblique view of all (temporalis, masseter, pterygoid) muscles loading regime of the power stroke of *Didelphis*, simulated to chew on beef (A), bone (B), modeled as a Triplet I (C) and modeled as full muscle activation (D). (E–H) Left oblique view of the absolute stress distribution resulting from a beef chewing muscle load (E), a bone chewing muscle load (F), a Triplet I muscle load (G) and a full muscle activation muscle load (H). (I–K) Load paths generated by different muscle loading regimes in left oblique view (I), right oblique view (J) and dorsal view (K). Vector width in A–D is scaled by muscle force magnitude. Loading the mandible with different muscle forces does not have noticeable effects on load path geometry.

**Fig. 9.**
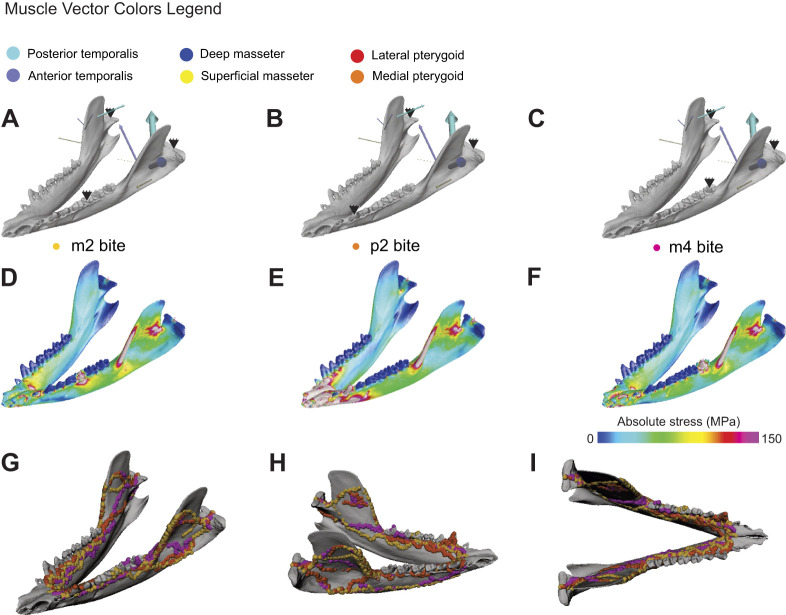
**Sensitivity analysis of the effect of alternative bite points on load path geometry on the cortical-trabecular model.** (A–C) Left oblique view of the full (temporalis, masseter, pterygoid) muscle loading regime of the power stroke of *Didelphis* chewing on beef at an m2 bite point (A), a p2 bite point (B) and an m4 bite point. (D–F) Left oblique view of the absolute stress distribution on muscle load in *Didelphis* chewing on beef with an m2 bite point (D), a p2 bite point (E) and an m4 bite point. (G) Left oblique view of load paths generated by different bite points. (H) Right oblique view of load paths generated by different bite points. (I) Dorsal view of load paths generated by different bite points. Vector width in A–C is scaled by muscle force magnitude. Loading the mandible with different bite point does not have noticeable effects on load path geometry.

Sensitivity analyses of loading conditions demonstrate that load paths are stable across different loading and constraining regimes (Figs 8 and 9). In all loading conditions, the cortical-trabecular model exhibits the same high stress regions on the ectocoronoid ridge and the masseter shelf (Figs 8E–H and 9D–F). Applying different external jaw muscle loads to the cortical-trabecular model shows no significant impact on load path geometry (Fig. 8I–K). Similarly, changing the bite point to different locations along the tooth row (respectively at p2, m2 and m4) does not yield noticeable changes in load path geometry (Fig. 9G–I). These analyses show that load paths are robust to changes in external loading regime.

## DISCUSSION

Load path analysis is a useful engineering technique to estimate force transfer, but it has yet to be used for testing hypotheses about mandible function. Here, we applied this novel method to address the long-standing question of the relative importance of cortical and trabecular bone in transmitting force between muscle attachment areas, bite points and the jaw joint. We created three mandible models of the opossum with alternative bone geometries of the interior ‘spaces’ (Fig. 1). These models are also informed by *in vivo* muscle functional patterns and tested by *in vivo* data on strain and bite force (Thomason et al., 1990; Crompton, 1995) (Fig. 3). We show that the interior model with trabecular bone tissue generates FE results that more accurately reflect *in vivo* bone strain and bite force magnitudes (Thomason et al., 1990; Crompton, 1995). Qualitatively, the deformation of all three models approximated *in vivo* chewing kinematics (Stilson et al., 2023). The cortical-trabecular model has the best matches with the *in vivo* axial strain regimes recorded with single element strain gauges (Crompton, 1995) (Fig. 4C). However, all models overestimated the bite forces expected of an opossum of similar size (Fig. 4D), with the cortical-hollow model deviating furthest from the expectations of the *in vivo* bite force data (Thomason et al., 1990). We hypothesize that differences in bite force between our models and the *in vivo* data may be due to errors in estimating muscle force (physiological cross-sectional area or jaw muscle activation). It is possible that the use of 0.3 N mm^−2^, as adopted from Hieronymus (2006), may overestimate the specific tension of opossum jaw muscles (Holmes and Taylor, 2021).

The inclusion of trabecular bone has important impacts on model results. The model with trabecular bone had the lowest bite force (and the most similar to the *in vivo* data) and overall lower strain and stress magnitudes than the other two models (Figs 3 and 4). The cortical-trabecular model also had the most consistent load paths from working side jaw muscles, but the least consistent from balancing side jaw muscles (Figs 5 and 6; Fig. S5). Most importantly, the load paths never enter the trabecular bone in this model (Figs 5,7–9). These results are consistent with the hypothesis that trabecular struts increase the stiffness of surrounding elements, as suggested by the early studies on bone functional adaptation by Roux (1881, 1885, 1895), as well as from more recent studies (e.g. Novitskaya et al., 2017; Mielke and Nyakatura, 2019; Williams et al., 2021). In particular, the solid and cortical-trabecular models in our analyses show similarities to the findings of Mielke and Nyakatura (2019) wherein a solid-cortical bone model and a fully trabeculated model yield grossly similar results. Inclusion of trabecular bone, when appropriate, is necessary for improving the precision in the modeling of the jaw of *Didelphis* and possibly other mammals.

Despite these differences in stress and strain regimes and load path geometry revealed by finite element analysis, the role of trabecular bone in force transfer is still poorly understood. Our data support the hypothesis that cortical bone, especially thickened surface features such as the anterior ridge of the ramus, the endocondylar ridge and masseteric shelf, are the primary load paths for transferring force (Figs 5,7–9) (Panagiotopoulou et al., 2020). Our data do not support the historical hypotheses that trabecular trajectories in the interior of bones are load paths for transferring force (Wolff, 1869). The primary load paths are in the cortical bone of the periphery in the cortical-trabecular model of the mandible. The load paths do not enter the trabecular bone. This is especially noteworthy given that, by simulating the filling of the mandibular canal with trabecular bone tissue (Fig. 1B), our cortical-trabecular model overestimates the amount of trabecular tissue inside the mandible. If the load path does not enter the trabecular bone in such an overestimated model, it is unlikely to enter the normally distributed trabecular bone of a wild-type mandible.

Our sensitivity analysis of periodontal ligament yields nuanced results. The inclusion of periodontal ligament has no effects on load path geometry, but presence of periodontal ligament increases the forces transmitted along the load path (Fig. 7). The lack of difference in load path geometry between the fused tooth roots and sockets (by simulation) and the presence of periodontal ligament between roots and sockets can be explained by Saint-Venant's Principle, which states that the further away a given element is from an artifact in loading, the less error that artifact will have on the given element (von Mises, 1945). Because the periodontal ligament is thin, modeling it will have limited effects on the rest of the mandible. Other studies examining the effects of modeling periodontal ligament have come to similar conclusions, noting that differences in strain regime only occur near the tooth sockets (Wood et al., 2011; Abraha et al., 2019). Because the load path results are essentially filtered FEA results, if the FEA is not sensitive to the inclusion of a material, the load path analysis should not be affected either. Remarkably, modeling periodontal ligament did influence the forces being transmitted along the load path. We hypothesize that this is the result of periodontal ligament increasing stress in the alveolar process (e.g. Daegling et al., 2008; Abraha et al., 2019; McGraw and Daegling, 2020). While omitting the periodontal ligament will not affect the studies of the location of the load path, researchers interested in calculating the forces transmitted along the load path in mandibles should also model periodontal ligament for more accurate results.

**Fig. 10.**
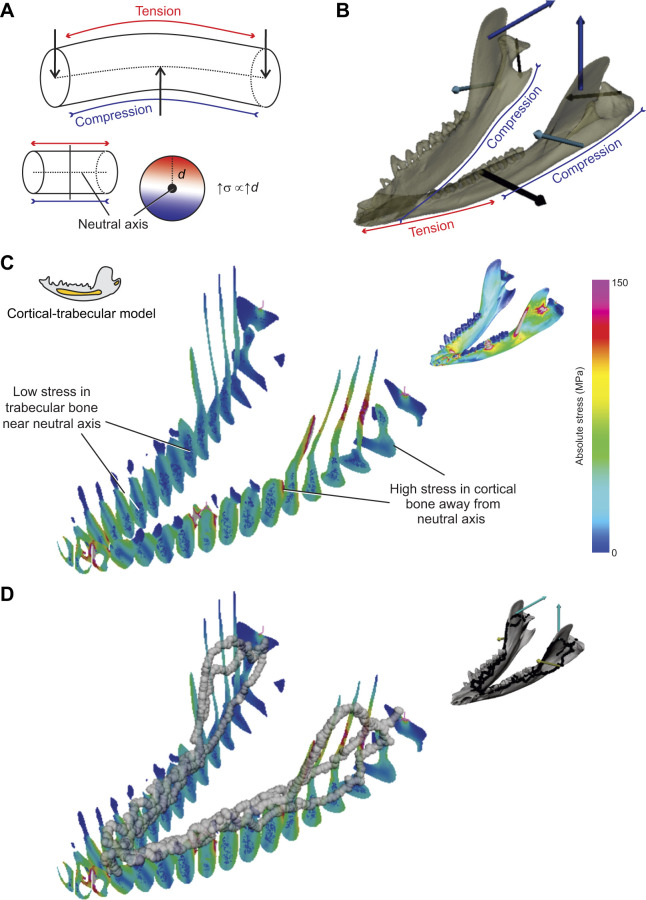
**Illustration of the influence of beam-bending mechanics of stress and load path location in the mandible of the trabecular (best validated) model.** (A) The four point bending regime of the *Didelphis* mandible hypothesized by Crompton (1995) and supported by validation against the *in vivo* results. (B) The generalized relationship between cross-sectional geometry and stress distributions in a prismatic beam in three-point bending: stress (ρ) is proportional (∝) to the distance away from the neutral axis (*d*). (C) Absolute stress distributions in coronal cross-sections of the trabecular bone model. (D) Absolute stress distributions in coronal cross-sections of the trabecular bone model with load paths overlaid. Bending mechanics predict that stress increases with distance from the neutral axis in a section. High stress concentrations on the mandible are found on the periphery of cortical bone. The continuous series of these stress concentrations is defined as the load path.

The restriction of the load paths to the cortical bone is understandable and mathematically predictable when one considers load path theory and cross-sectional geometry (Fig. 10). Load paths follow the shortest, stiffest route (Kelly et al., 2011; Wang et al., 2017; Zhao et al., 2021) and cortical bone is 20–30% stiffer than trabecular bone (Beaupré et al., 2000). This means that if the load path would go through the trabecular bone, such ‘trabecular’ load path would have to be at least 20–30% shorter to balance it for its lower stiffness, and even under this scenario, the optimization relationship between stiffness and length may not be proportional for a load path. Moreover, bending stresses in a beam increase as distance away from the neutral axis increases (Vogel, 1988; Fig. 10A). Therefore, the continuous series of highest stressed elements should be expected to be on the periphery of the mandibular corpus, not in the trabecular bone near the neutral axis of loading (Fig. 10). These factors ought to restrict the load paths to the cortical bone in bony elements that can be modeled as a beam, as is observed here. These observations do not imply that trabecular bone is not loaded and without function, as trabecular bone is known to correlate with a variety of behavioral and ecological specializations (e.g. Skinner et al., 2015; Mielke et al., 2018; Tsegai et al., 2018; Ryan et al., 2018; Amson and Kilbourne, 2019; Saers et al., 2020; Chirchir et al., 2022) and can respond to loading conditions (e.g. Biewener et al., 1996; Barak et al., 2011; Saers et al., 2022). However, interpretation of trabecular bone patterns should be performed with caution (Bertram and Swartz, 1991; Cowin, 1997, 2001; Skedros and Baucom, 2007; Sinclair et al., 2013): we need a better understanding, in the context of the whole bone, how trabecular bone is stressed and strained, how it transmits load, and the process of trabecular bone replacement and remodeling in response to those loading conditions (Bertram and Swartz, 1991). Load path analysis and beam mechanics suggest that trabecular bone is unlikely to be the primary load path through long bones with a ‘thick’ cortical bone shell. Exactly how variation in cortical bone thickness impacts variation in location of the primary load path remains to be shown: we hypothesize that, in bone regions where cortical bone thickness approaches that of average trabecular thickness, such as mammal tarsal bones (Tsegai et al., 2018), the load path is more likely to run through trabecular bone. While we acknowledge the functional significance of trabecular bone, caution is warranted in interpreting its role in load transmission, especially as it applies to trajectory theory.

Load path analysis offers new insights into mandible function. In the tetrapod mandible, forces are transferred from input jaw muscle forces to the bite point, but only forces parallel to the load path are transmitted along it (Zhao et al., 2013, 2021) (Fig. S4). The force components orthogonal to the load path generate bending moments and shear forces on the bone on and near the load path (e.g. Singh, 1996). These bending moments and shear forces will increase in mammal mandibles with tall rami – adaptations for increasing attachment areas and moment arms of the jaw adductors – because the load paths through these shapes must curve to pass from muscle insertions to jaw joints and biting teeth. This not only results in inefficiency by ‘wasting’ force on bending moments and shear, rather than transmitting that force to the bite point, but it suggests that increasing jaw adductor size through increases in ramus height must be accompanied by proportionate increases in bony structures resistant to bending and shear, especially in areas where load paths show the greatest curvature, such as the corpus–ramus junction and the symphyseal region (e.g. Smith et al., 2021).

### Relevance for experimental biology

We suggest that load path analysis is a promising new tool for biologists who use both modeling (theoretical) and *in vivo* approaches to understand vertebrate functional morphology. Load path analysis can highlight regions of skeletal structures where bone distribution are closely related to stress and strain regimes, such as areas where load paths curve or where local bone protuberances or cortical bone thickenings – such as the endocondylar and endocoronoid ridges – stiffen otherwise thin bony plates (Weidenreich, 1939; Panagiotopoulou et al., 2020). Load path analysis is a new method for evaluating the functional and structural reality of trajectories of force transfer in the mandible, as postulated by Walkhoff (1902) but never fully resolved, and also in the cranium (see Prado et al., 2016, for a review). Moreover, load path analysis provides a way to demonstrate a hypothesized relationship between a given load and a trabecular pattern. An understanding of load paths may lead to new questions about plasticity in response to perturbations of, for example, diet and feeding behavior. Are load paths a constraint on plasticity, or a mechanistic driver of plasticity? Finally, load paths would be informative for selection experiments by demonstrating which parts of the skeletal structure are actually transmitting force. Given that the study of functional bone adaptation is at its core an argument for optimization of material around trajectories (Cullman, 1866; von Meyer, 1867; Wolff, 1869; Bertram and Swartz, 1991; Ruff et al., 2006; Skedros and Baucom, 2007) and load path analysis is often employed to optimize use of material (e.g. Sakurai et al., 2003; Bassandeh, 2012; Kelly et al., 2019), deploying load path analysis in these experimental contexts will provide new insights into bone biology and functional adaptation. The data collected through load path analysis provide new predictions for experimental studies.

The discrepancy between the cortical-trabecular cortical model and *in vivo* strain magnitudes highlights the need for renewed efforts in experimental research of the mechanics of mandibles with unfused mandibular symphyses. The lack of information on the exact location of strain gauges from Crompton (1995) hinders a precise comparison of our model data to *in vivo* loading. Furthermore, there is a dearth of data on the stiffness of an unfused mandibular symphysis and how the different tissues of the mandibular symphysis contribute to force transfer across the symphysis (e.g. Scapino, 1965, 1981; Beecher, 1979). Having these data is key to creating a comparative framework of gnathostome feeding mechanics beyond anthropoid primates. Research on the *in vivo* loading and structure of unfused mandibular symphyses are necessary to understand the evolution of mandibles (e.g. Vinyard and Ravosa, 1999; Lieberman and Crompton, 2000; Gill et al., 2014; Lautenschlager et al., 2018; Smith et al., 2021) and for validation of animal models for mandible biomedical research.

### Conclusion

Classic models of bony trajectories have long suggested that trabecular tissue is an important route for force transfer through bones (Cullman, 1866; von Meyer, 1867; Wolff, 1869; Walkhoff, 1902). Although the trabecular trajectory model implicitly invokes load paths through trabeculae, this has, until the present paper, not been tested. Here, we investigated the role of trabecular bone in the behavior of FEMs of the opossum mandible and on the location of the load paths of the mandible. Trabecular bone lowers strain magnitudes and bite force and increases consistency of load paths in the cortical bone of the mandibular corpus. Despite these trends, the primary load path stays in the stiffest cortical bone, and does not enter the trabeculae, which are less stiff. While we are not arguing that trabecular bone is not loaded, strained, stressed and responsive to changing loads, our results do show that trabecular bone does not function as the primary load path in these models. These results run counter to historical ideas that trabecular ‘trajectories’ mark the location of force transfer. If trajectories have structural reality, or morphological manifestations (Prado et al., 2018), they are most likely to be found in cortical bone. In this respect, load path theory offers new ways to study form–function relationships in vertebrate jaws, and is a promising tool for future investigations into comparative functional morphology of the skeletal system.

## Supplementary Material

10.1242/jexbio.247030_sup1 Supplementary information

Dataset S1. Contains tables of muscle modeling parameters and material properties used for the models.
