# Supplementary material for: A new biomechanical model of the mammal jaw based on load path analysis
Source: J Exp Biol. 2024 Sep 30;227(18):jeb247030. doi: 10.1242/jeb.247030 (PMC11463961; doi:10.1242/jeb.247030)
Supplement: Supplementary information [file jexbio-227-247030-s1.pdf]

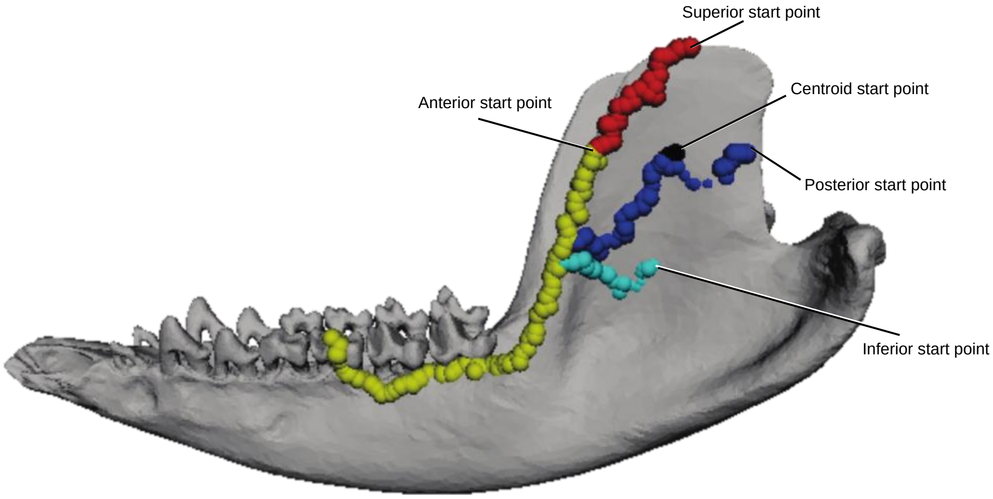

Fig. S1. Sensitivity analysis of different starting points for the working temporalis load path to the bite point. Different start points from different borders of the temporalis attachment on the ramus ultimately create the same load path.

Journal of Experimental Biology • Supplementary information

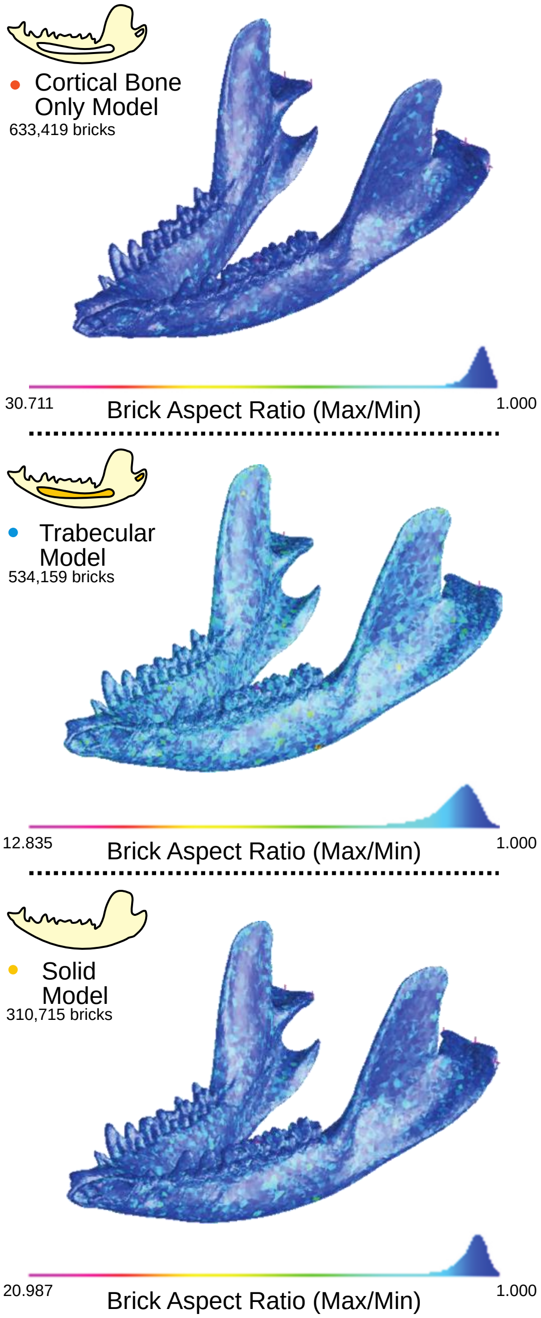

Fig. S2. Distribution of brick element aspect ratios in all three alternative models. A. Simplified hollow model. B. Trabecular model. C. Solid model. High aspect ratios could indicate invalid forces in the load path. Although all models have a relatively long tail on the distribution of high aspect ratio brick elements, the vast majority of bricks are skewed towards 1.00, indicating invalid forces are not a concern for these models as the high aspect ratio bricks are likely not in the load path.

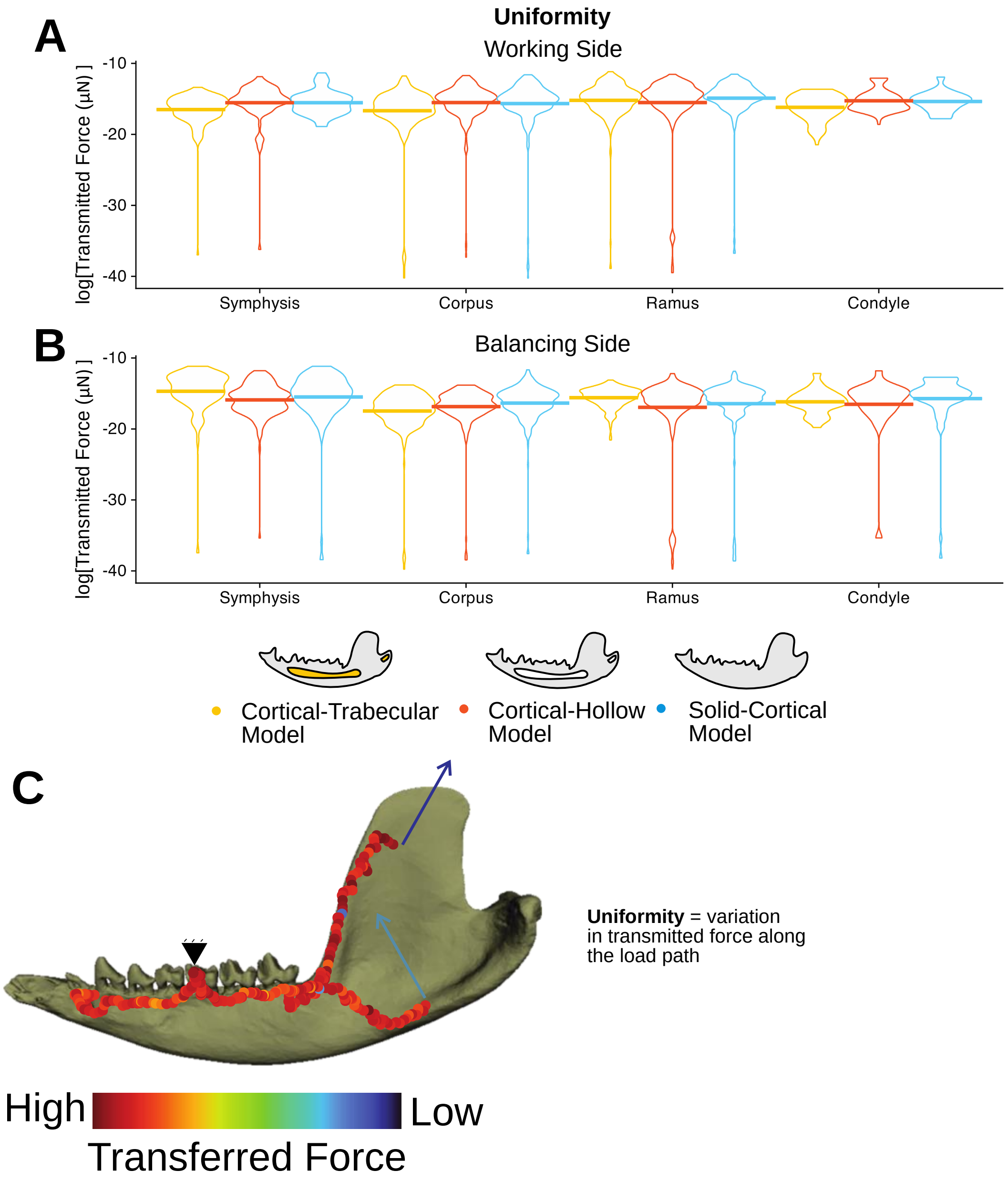

Fig. S3. Distribution of forces transmitted parallel to the load path among the three models. **A.** Forces transmitted parallel to the load path among on the three models on the working side hemimandible. **B.** Forces transmitted parallel to the load path among on the three models on the balancing side hemimandible. **C.** Schematic color gradient of transferred force in the mandible illustrating the concept of uniformity. In all three models, forces transmitted parallel to the load path are low and have a wide distribution. Transmitted forces are similar in all regions of the mandible. None of the three models has significantly better uniformity of force transmitted parallel to the loadpath.

Journal of Experimental Biology • Supplementary information

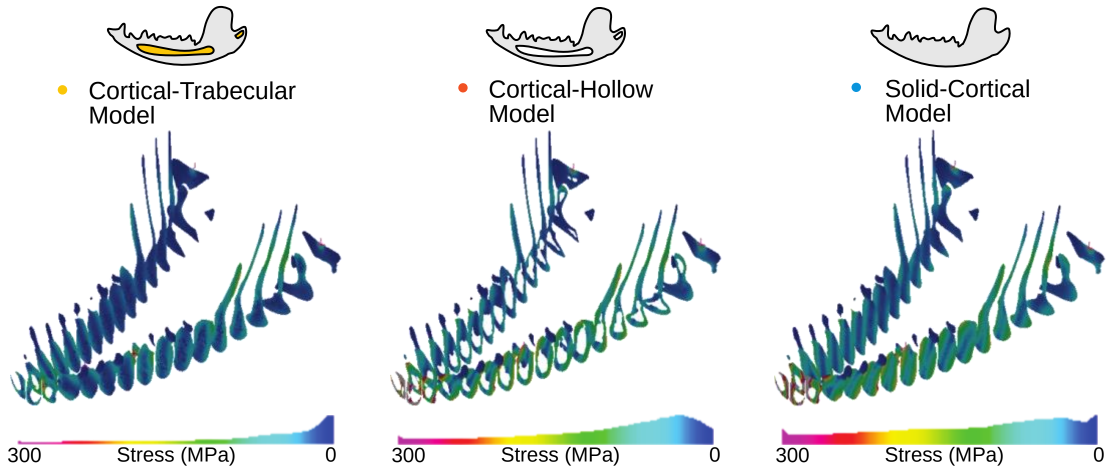

Fig. S4. Cross-sections and histogram of absolute stress distribution in the three interior bone geometry models. The cortical-trabecular model has on average lower stress magnitudes than the other two models.

Journal of Experimental Biology • Supplementary information

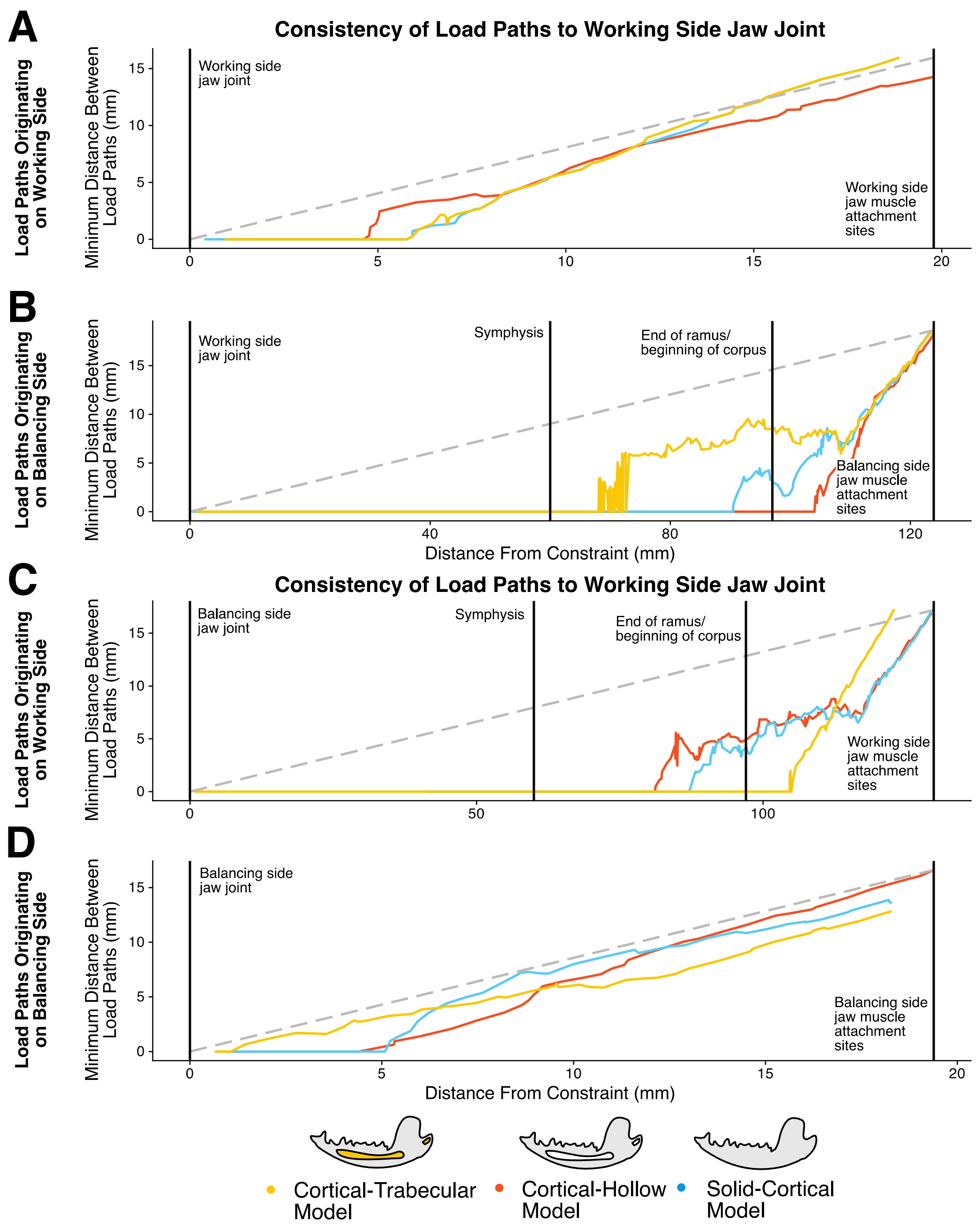

Fig. S5. Consistency of load paths to working side and balancing side jaw joints. **A.** consistency of load paths originating from the working side hemimandible to the working side jaw joint. **B.** consistency of load paths originating from the balancing side hemimandible to the working side jaw joint. **C.** consistency of load paths originating from the working side hemimandible to the balancing side jaw joint. **D.** consistency of load paths originating from the balancing side hemimandible to the balancing side jaw joint.

Journal of Experimental Biology • Supplementary information

Dataset S1. Contains tables of muscle modeling parameters and material properties used for the models.

Available for download at
https://journals.biologists.com/jeb/article-lookup/doi/10.1242/jeb.247030#supplementary-data
